# Injury as a Result of Children and Adolescent Labor—An Association with Ethnicity and Peripherality: A Retrospective Cohort Study Based on the Israeli Trauma Registry

**DOI:** 10.3390/ejihpe14010009

**Published:** 2023-12-31

**Authors:** Bella Savitsky, Irina Radomislensky, Eldad Katorza, Arielle Kaim

**Affiliations:** 1 Department of Nursing, School of Health Sciences, Ashkelon Academic College, Yitshak Ben Zvi 12, Ashkelon 7821100, Israel; 2 Israel National Center for Trauma & Emergency Medicine Research, The Gertner Institute for Epidemiology and Health Policy Research, Sheba Medical Center, Tel-Hashomer, Ramat Gan 5262100, Israel; irinar@gertner.health.gov.il (I.R.); eldad.katorza@sheba.health.gov.il (E.K.); 3 School of Medicine, Tel Aviv University, Tel Aviv 6139001, Israel; 4 Antenatal Diagnostic Unit, Department of Obstetrics and Gynecology, Chaim Sheba Medical Center, Tel-Hashomer, Ramat Gan 5262100, Israel; 5 Arrow Program for Medical Research Education, Chaim Sheba Medical Center, Tel-Hashomer, Ramat Gan 5262100, Israel; 6 Department of Emergency and Disaster Management, School of Public Health, Faculty of Medicine, Tel Aviv University, Tel Aviv 6139001, Israel; akaim@tauex.tau.ac.il

**Keywords:** children, adolescents, employment, occupational, injury, work-related, hospitalization

## Abstract

Background: Working children and adolescents face a heightened risk of work-related injuries. This research aimed to assess the rate of hospitalizations resulting from work-related injuries among children and adolescents in Israel, with a specific focus on disadvantaged populations. Methods: This nationwide retrospective cohort study utilized The Israeli National Trauma Registry (INTR). It included 642 children and adolescents aged 13–17 hospitalized due to work-related injuries from 2015–2022. Results: Arab children had over five times the risk of hospitalization due to work-related injuries compared to Jewish (RR = 5.5, 95% CI: 4.7–7.4). Despite the 2018 law prohibiting young people from entering this type of work, the most common type of work leading to hospitalization was construction, accounting for 40.2% of Arab and 11.9% of Jewish injuries (*p* < 0.001). After adjustment, road traffic accidents and falls presented the highest odds of at least severe injury. Arabs had three times significantly higher odds of at least moderate injury compared to Jews. Conclusions: Prioritizing the creation of safe job opportunities for Arab teenagers is imperative. Strict enforcement measures, particularly within the construction industry, especially among Arab youth and during night shifts, are essential. These initiatives should focus on establishing secure and sustainable employment opportunities for children and young individuals, effectively reducing the risks associated with hazardous labor practices. In addition, the implementation of educational programs in the school curriculum covering essential aspects of youth employment is vital.

## 1. Background

The International Labor Organization (ILO) describes children’s or adolescents’ participation in the workforce as generally positive when this activity does not affect their health and personal development or interfere with their schooling. This is in line with the human capital investment approach, which is defined as the process of developing and enhancing the knowledge, skills, and abilities of individuals, which in turn contributes to their productivity and earning capacity in the future [1,2]. Child labor is defined as work prior to the age of 14. Globally, 186.3 million children aged 5–14 work worldwide, mostly in developing countries [3]. In developed countries, employment under the age of 14 is forbidden [4,5]. ILO Convention No. 138 establishes that “employment of adolescents is allowed at the age of 15, with the possibility to temporarily set the general minimum age at 14 for countries whose economy and educational facilities are insufficiently developed” [1]. The main concern of public health practitioners is that most employed children and adolescents work under circumstances that jeopardize their health [4], where work-related injury is one of the potential consequences [6]. Scholars argue that engaging in work during adolescence can foster positive growth in young individuals when the work is of moderate intensity, consistent in duration, and balanced with their academic commitments according to the principles of the human capital approach [7,8]. Consistent employment can aid in cultivating time management abilities, which can be valuable as young individuals transition into college. This is especially relevant because many college students continue to work to sustain themselves and contribute to their tuition expenses, even if only partially [6].

Conversely, young employees face an elevated risk of work-related injuries due to several factors associated with their jobs, including workplace hazards, violations of child labor laws, the rapid pace of work, minority status, and a lack of skills, experience, supervision, and comprehensive safety training [9,10]. Additionally, young workers may be less likely to identify workplace hazards, speak up about safety concerns, and be knowledgeable about their legal protections [10]. As a result, national statistics from five Nordic countries demonstrate that young workers are nearly twice as likely to experience workplace accidents compared to their older counterparts. Furthermore, they are exposed to potentially harmful conditions and hazardous tasks to a greater extent than older employees [11].

In developed countries, special concern is associated with child labor among ethnic minorities. Gypsies in Europe, Aboriginal people in Australia, Inuits in Canada, Blacks and Latinos in the United States, and migrants represent populations at risk for children’s employment [12].

In Israel, almost 10% of adolescents aged 15–17 worked during 2017, according to data from the Central Bureau of Statistics [13]. This data likely reflects a lower estimate of the actual percentage of employed adolescents. In a 2014 survey, 31% of adolescents aged 14–17 disclosed that they had been employed at some point during the year [14]. According to the survey conducted in 2014 by the Ministry of Economy, the most frequent place of work for adolescents was the food industry (waiting or kitchen work)—this sector employed 36% of working adolescents (43% of boys and 20% of girls); 15% worked supplying services of babysitting and baby care (39% of girls and 2% of boys), 12% worked in retail sector (as sellers or cashiers), 8% gave private lessons, almost 6% worked in delivery service and 6% worked in construction [14]. As indicated by 53% of the sample, the primary motivation for commencing work was the necessity to earn their own income. Additionally, 22% cited the need to assist their families, 14% expressed a desire to gain occupational experience, and 9% mentioned that they began working out of boredom [14].

Occupational opportunities are influenced by the geographical location of workers. In Israel, Arab communities are predominantly located in geographic peripheries. Being remote from economic hubs often results in fewer chances for these communities to blend into the broader job market. This scenario particularly affects the mobility of women and younger workers [15]. The scarcity of employment options in these outlying regions forces young workers to accept less favorable employment conditions, which employers may manipulate [13].

In Israel, the legislation aims to safeguard young workers from exploitation and potential health risks. One instance of such legislation is the Youth Employment Law, which prohibits the employment of individuals under the age of fifteen. Moreover, this law also restricts minors from engaging in specific work activities and certain work environments. For instance, since 2018, employing young people in tasks related to building, construction, or construction activities on a construction site has been illegal. The law further prohibits a minor from engaging in night work between 8 p.m. and 8 a.m. for minors to whom the Compulsory Education Law applies and between 10 p.m. and 6 a.m. for those to whom it does not [16]. These limitations are frequently breached, primarily due to employers not obeying the law and partly also due to the significant number of young workers (44%) who are unaware of their rights. In the survey conducted among young workers, it was found that 72% of them did not receive any training related to occupational safety, and 27% reported experiencing injuries either during work or while commuting to work [17].

Regarding the prevalence of work-related injuries among adolescents in Israel, it is worth noting that nearly a thousand teenagers sought medical attention in the Emergency Department (ED) annually due to workplace injuries between 2011 and 2013 [18].

The aim of this research was to illuminate the extent of hospitalizations resulting from work-related injuries among children and adolescents in Israel, with a specific focus on disadvantaged populations like ethnic minorities. The study aimed to identify the group most vulnerable to severe injuries and evaluate how recent legislation has impacted trends in hospitalizations related to work-related injuries.

## 2. Methods

### 2.1. Study Design

This is a retrospective cohort study based on the Israeli National Trauma Registry (INTR) in the period between 1 January 2015 and 31 December 2022. The INTR provides comprehensive data on hospitalized trauma patients from all six Level I Trauma Centers (TC) and 14 of the 20 Level II TCs in Israel. All hospitalized trauma patients classified with an ICD-9-CM diagnosis code 800–989.9 who were admitted to the Department of Emergency Medicine (ER), hospitalized, died in the ER, or were transferred to or from another hospital are included in the database. The registry does not include poisoning, chemical asphyxiation, inhalation injuries, drowning, and choking; casualties who died at the scene of the event or on the way to the hospital; and admissions 72 or more hours following the event. The data is recorded by trained trauma registrars at each TC under the supervision of a trauma director. Electronic files are transferred to the INTR, where quality assurance is carried out prior to data analysis.

### 2.2. Study Population

The study population included 642 children and adolescents, Israeli citizens aged 13–17 hospitalized due to work-related injuries.

### 2.3. Study Variables

Age was used as a continuous variable. Gender: male/female. Population group: Jews/Arabs.

Peripherality index of local authorities: The peripherality index characterizes and classifies localities and local authorities in Israel according to their geographic location relative to population centers. The Central Bureau of Statistics (CBS) devised this index. It categorizes localities and local authorities into ten clusters based on their index value. These clusters range from cluster 1, which encompasses the most geographically remote units, to cluster 10, which includes the most centrally located units. The classification of peripheral clusters was determined using 25% percentiles as follows: clusters 1 to 4 are considered the most peripheral, cluster 5 is peripheral, clusters 6 and 7 are central, and clusters 8 and above are classified as very central [19].

Injury mechanisms were categorized as Road Traffic Crashes (RTC), falls, burns, cuts and lacerations, violence, struck by an object, and unknown.

The type of work was identified by searching the free text of the injury description and categorized: food industry, building and reconstruction, agriculture, delivery, garage, retail sector, and others. Hour of the work incident: night work (10 p.m.–6 a.m.)/non-night work.

Injury Severity Score (ISS)—the sum of the squares of the single highest Abbreviated Injury Scale score for each of the three most severely injured body regions [20] categorized 1–8 (mild injury); 9–14 (moderate injury); 16+ (severe injury) and 25+ (critical injury) [21]. Length of Hospital Stay (LOS) was used as a continuous variable. Intensive Care Unit (ICU) stay: yes/no. Operation during the hospitalization: yes/no.

### 2.4. Statistical Analysis

A univariate analysis examined the association between population group and injury characteristics using the χ^2^ test and Mann–Whitney non-parametric test for differences in age and LOS, which is not normally distributed.

The risk of being hospitalized following work-related injury was calculated using the data on the Israeli population of children and adolescents in 2018, according to the Central Bureau of Statistics (2,130,700 Jewish and 650,660 Arab children aged 0–17 years) [22].

Multivariable analysis with a logistic regression approach was used, with the dependent variable being the occurrence of at least moderate injury (ISS 9+). The independent variables considered in the analysis included age, population group, peripherality index, trauma type, and the mechanism of injury. Multicollinearity between all variables included in the multivariable analysis was assessed using Variance Inflation Factors (VIF), and the maximum VIF was 1.2. Analyses were carried out using SAS V.9.4 statistical software. For all analyses performed, a value of *p* < 0.05 was considered statistically significant.

## 3. Results

### 3.1. Demographic Characteristics of the Study Population

During the study period, a total of 642 children and adolescents aged 13–17 were hospitalized due to work-related injury; of them, 38.0% (n = 244) were Jewish, and 62.0% (n = 398) were Arabs. The median age was similar among Arabs and Jews (17 years old). Most of the hospitalized were teens (14–17 years old). Out of the 13 children who were hospitalized while they were at the age of 13, eleven of them were of Arab ethnicity.

Boys accounted for the majority of those hospitalized (92.5%). However, among the Arab population, the proportion of boys was significantly higher compared to the Jewish population (96.7% vs. 85.7%, *p* < 0.001).

Arab individuals predominantly resided in more peripheral regions, with 58.3% living in the most peripheral areas, in contrast to 23.1% among Jewish individuals. In the case of Jewish children and adolescents, 37.6% lived in the most central areas, while only 13.3% of Arab children and adolescents did so (*p* < 0.001). See Table 1 for additional details.

Over the duration of the study, no significant demographic changes were observed among the hospitalized individuals.

**Figure 1:**
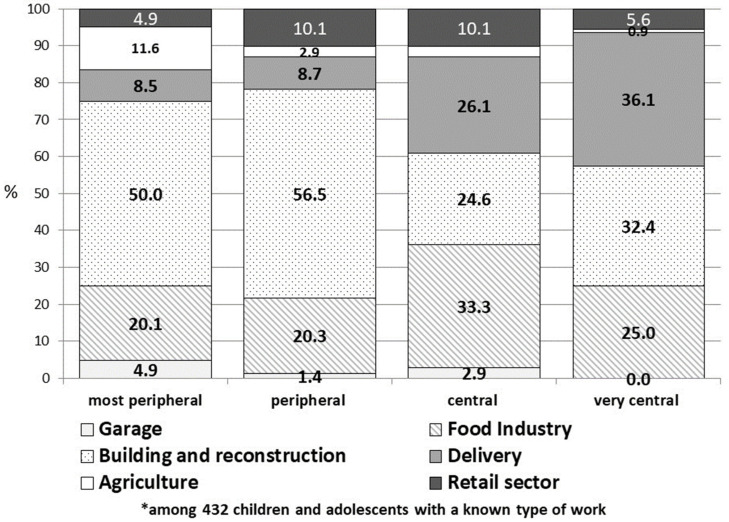
Distribution of work’ types* by peripherality of the area.

Figure 1 illustrates the distribution of work types according to peripherality. Among children and adolescents hailing from peripheral regions, a greater percentage experienced injuries in the building and reconstruction sector (50.0% in the most peripheral areas and 56.5% in peripheral areas), as opposed to those from central and very central areas (24.6% and 32.4% respectively). Conversely, children and adolescents originating from more central areas had a higher proportion of injuries related to delivery (26.1% and 36.1%) and the food industry (33.3% and 25.0%) compared to the lower proportion of these work types in peripheral areas.

### 3.2. Accident Characteristics

**Figure 2:**
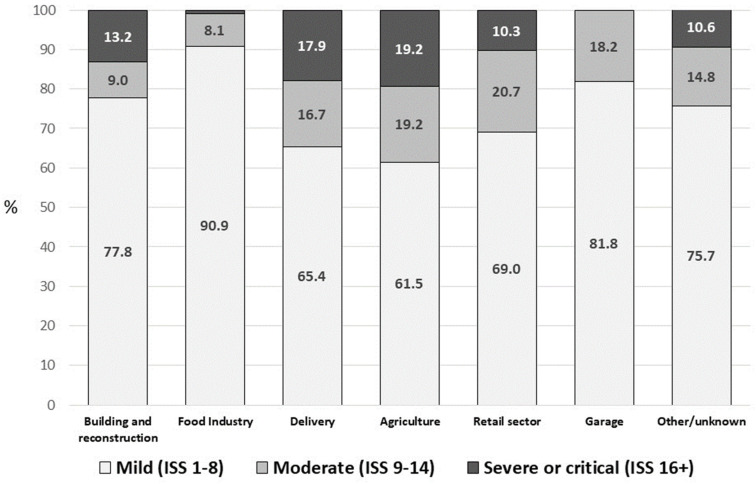
Injury severity by the work type.

In 32.9% of instances, it was not possible to determine the type of work through free text analysis. The rate of unidentifiable work types was notably higher among girls, with 43.8% of cases lacking clear information regarding the specific type of work responsible for their injuries. There was no change in the proportion of unknown types of work during the study period. An upward trend was observed in the frequency of injury in the food industry (29.7% in 2022 vs. 16.7% in 2015), and the opposite trend was observed in the frequency of injury in building or construction (21.9% in 2022 vs. 34.3% in 2015) (*p =* 0.011). The severity of the injury by work type is depicted in Figure 2.

Among cases where the type of work could be identified, the most common type of work at the time of the accident resulting in hospitalization was building or reconstruction work, which accounted for injuries in 29.4% of children and adolescents (40.2% among Arabs and 11.9% among Jews, *p* < 0.001). The legislation prohibiting the employment of children in the building and construction sector was enacted in 2018 [23]. Prior to the year of legislation (2018), the number of hospitalizations resulting from work at building or reconstruction was as follows: 35 in 2015, 34 in 2016, and 37 in 2017. Subsequently, there was a significant drop in these numbers in 2018 and 2019, with 19 cases each year. This was followed by a gradual decline in the subsequent years, with the number of cases decreasing from 16 in 2020 to 14 in 2022.

A significant proportion of these children and adolescents sustained injuries as a result of falling from heights, accounting for 34.9% of the cases (50.0% among Arabs and 33.6% among Jews, *p* < 0.001). Those who were injured while engaged in building and reconstruction work exhibited a notable incidence of at least moderate injuries, with 9% experiencing moderate injuries (with an ISS score of 9–14) and 13.2% suffering severe injuries (with an ISS score of 16+).

The second most common type of work at the time of the injury was in the food industry, accounting for 15.4% of cases. Notably, this particular field of work was more prevalent among hospitalized girls, with 27.1% of girls in the sample being employed in the food industry. Among these young workers, 42.4% were injured due to cuts or lacerations, 21.2% were burned, and 12.1% were injured while using kitchen equipment. Injuries in the food industry were less severe (only 9.1% sustained at least moderate injuries with ISS 9+).

The third frequent type of work was delivery. These young workers were injured in 97.4% of cases of the Road Traffic Crashes (RTC) while using motorcycles, scooters, and electric bikes. The injury of these children and adolescents had moderate severity (ISS 9–14) in 16.7% of cases and in 17.9%—severe (ISS 16+) injury.

A small proportion (4.5%) of hospitalized children and adolescents were injured while working in the retail sector. The injuries primarily occurred due to incidents such as heavy objects falling within a warehouse or when individuals were loading items onto shelves. Additionally, 4.0% of injuries were associated with agricultural work, with the main causes being similar to those in the retail sector or involving falls from heights while carrying out tasks like tree pruning. The injury of these children and adolescents had moderate severity (ISS 9–14) in 19.2% of cases and in 19.2%—severe (ISS 16+).

A minority of individuals, all of whom were of Arab descent, sustained injuries while working in garages, primarily due to incidents involving objects striking them.

Arabs had a significantly higher prevalence of at least moderate injuries (ISS of 9 and above) in comparison with Jews (26.6% vs. 18.0%) (*p* = 0.013). All five children who died due to injuries were of Arab ethnicity.

The injury profiles of patients hospitalized over the course of the study did not show any significant differences.

### 3.3. Time of the Accident

Israeli legislation prohibits the employment of children and teenagers during the nighttime hours, specifically from 10 p.m. to 6 a.m. [24]. Nevertheless, 18.5% of the study sample (n = 119) experienced injuries during this period. A majority of the children and adolescents injured at night were of Jewish ethnicity (57.1% vs. 42.9%, *p* < 0.001). Among those injured during the night, 41.9% were employed in the food industry, 26.7% were engaged in food delivery, and 24.4% worked in the construction sector.

### 3.4. Probability of at Least Moderate Severity of Injury

This analysis was conducted only among 432 cases with identifiable types of work. Table 2 represents a univariate and multivariable model with at least moderate injury as a dependent variable.

In a multivariable logistic regression model (Model I, Table 2) with at least moderate injury as a dependent variable, the odds of Arabs sustaining at least moderate injury were almost three-fold higher than the odds of Jews (OR = 2.9, 95% CI: 1.4–6.3). Compared to the food industry, the highest odds for at least moderate injury had been among those engaging in delivery work (OR = 8.8, 95% CI: 3.3–22.9), followed by those partaking in work in the agriculture and retail sectors. Peripherality was not associated with the outcome. This model explained 12% of the variance in the probability of at least moderate injury.

After adding the mechanism of injury to the model (Model II, Table 2), the type of work was no longer associated with the outcome. In comparison with cuts and lacerations, those who were injured due to RTC or falls had more than fourteen-fold higher odds for at least severe injury. Injuries resulting from machinery were linked to over an eightfold increase in odds, while burns were associated with a fivefold higher likelihood, and incidents involving being struck by objects had odds of more than fourfold. Notably, in this model, the higher odds among Arabs compared to Jews remained consistent. This model explained 26% of the variance in the probability of at least moderate injury.

Sensitivity analysis was performed with all 642 cases, including hospitalized cases, whose type of work was unknown. This analysis yielded similar results.

The relative risk of being hospitalized during the study period was calculated using ordinary incidence rates of Jews and Arabs. During this period, 238 Jews and 398 Arabs were injured and hospitalized. As of 2018, which marked the midpoint of the study period, the population in Israel consisted of 2,130,700 Jewish children and adolescents, as well as 650,660 Arab children and adolescents. Thus, the risk of hospitalization stood at 11.2 per 100,000 for Jewish children and adolescents, while it was 61.2 per 100,000 for their Arab counterparts (RR = 5.5, 95% CI 4.7–7.4). Hence, Arab children and adolescents faced a risk of being hospitalized following a work-related injury that was five and a half times higher than that of Jewish children and adolescents.

## 4. Discussion

The objective of this study was to study the phenomenon of work-related injuries among children and adolescents, particularly in disadvantaged populations characterized by their ethnic background and peripheral location. The findings indicated that the primary risk factor for experiencing a more severe injury is the mechanism of injury itself. Occupations with distinct injury mechanisms tend to result in a higher number of hospitalized children and adolescents. For instance, in the case of individuals working in building and reconstruction sites, the primary injury mechanism is falling from heights, which accounts for the elevated severity of the injuries observed.

The study revealed that the risk of Arab children and adolescents being hospitalized following work-related injury is more than five-fold higher than the risk of their Jewish counterparts. Interestingly, this heightened risk among Arab individuals does not appear to be directly correlated with higher rates of employment among children and adolescents in this demographic. Recent data reveals that the employment rate for teenagers in the Arab sector is actually six times lower than that among Jewish youth [25]. Consequently, this increased risk among Arab youth seems to be indicative of their greater likelihood of experiencing injuries that necessitate hospitalization. Notably, the research data also indicated that hospitalized Arab children predominantly reside in peripheral regions of the country. Conversely, children and adolescents in the Arab sector may engage in compensated work without formal employment status. This phenomenon is further highlighted by a significant income disparity, where the gap between actual earnings and income reported to the Israeli Tax Authority can reach up to 26% in the Arab population [26].

In Israel, Arabs comprise 23% of the Israeli population aged 0–17 years old [22]. The majority of the Arab children (96%) are Muslims [22]. Arabs and Jews differ in religion, culture, and language. The Arab population lives in mostly all-Arab communities located in rural areas in Northern and Southern Israel [27]. When compared to the Jewish population, Israeli Arabs have lower income, less access to education, and higher unemployment rates [28]. Most Arab localities are situated in the country’s socioeconomic and geographic peripheries, factors that significantly impact their economic and social standing. According to data from the Central Bureau of Statistics, nearly all (95%) of Arab localities are categorized within Israel’s lowest socioeconomic brackets, with 11% of them falling into the lowest cluster (cluster 1). In stark contrast, only 5% of Arab localities are classified within clusters 6 to 10 [29]. In peripheral areas, there exists a substantial disparity between the availability of job opportunities for teenagers and the actual demand for such positions. Consequently, in these areas, a pronounced compromise is often observed among young individuals regarding their wages and employment terms, while employers may also exploit this situation to their advantage [13]. The findings of this study indicate that within these regions, a notably large percentage of Arab children and adolescents have sustained injuries while engaged in building and reconstruction work, with falls from heights being a prevalent cause of such accidents. An analysis of workplace injuries in the construction and rebuilding sectors showed that a greater percentage of Arab workers sustained injuries from falls at height compared to Jewish workers, indicating a higher incidence of involvement in high-risk occupations. Accidents involving falls from heights at construction sites frequently result in traumatic injuries, leading to significant mortality rates and imposing a substantial financial burden on healthcare systems [30]. In Japan, for example, it has been documented that falls from elevated positions constitute the second highest cause of trauma-related fatalities, trailing only behind motor vehicle collisions, and they represent the leading cause of trauma-related deaths among young individuals [31]. Hospitalization cases, much like the visible tip of an iceberg, exclusively pertain to the most serious traumatic incidents. This research has revealed that despite legal prohibitions against employing underage individuals in the construction and building industry, this practice persists, leading to annual instances of young workers requiring hospitalization due to workplace injuries [23]. Construction in Israel is a sector where Arab workers are frequently employed, contributing significantly to the country’s infrastructure development. However, it is also a field fraught with numerous safety challenges. Regrettably, Israel has witnessed alarmingly high rates of injuries and fatalities in the construction industry [32]. Annually, dozens of workers lose their lives as a result of hazardous working conditions. The mortality rate among workers on Israeli construction sites is 2.5 times higher than that in the European Union when considering fatalities per 100,000 workers, according to the Israeli Workers’ Hotline [33]. Work environments prone to falls, like construction zones, necessitate rigorous safeguards to either prevent falls or lessen the impact should they occur. The issue is exacerbated by the underreporting of accidents, weak enforcement, inadequate safety regulations, the absence of appropriate gear and supervision, deficient training, and the employment of unlicensed workers [34]. Strengthening the enforcement of existing legislation is crucial to addressing this issue. This requires a robust system of oversight and accountability, increased resources for regulatory agencies, and stringent penalties for non-compliance. Effective enforcement will ensure that legal prohibitions against employing underage individuals in the construction industry are not merely symbolic but actively prevent exploitation and safeguard young workers from harm.

The current study highlighted that among the hospitalized, Arabs have higher odds for more severe injury, and this finding is independent of the work type and injury mechanism. This finding is in line with previous work published, which found that Arab children have a higher ISS; however, the odds ratio of proceeding directly to the hospital was lower for Arab children compared to Jewish children, controlling for injury severity [35].

Another occupation within the study population that exhibited a significant prevalence of injuries is employment in the delivery sector. Among this group of workers, two-wheeled food delivery drivers, including cyclists and scooter riders, emerge as the most susceptible and at-risk individuals on the road. The mode of transportation used in the delivery sector plays a pivotal role in understanding this higher susceptibility to injuries among its workers [36]. Two-wheeled food delivery drivers frequently navigate in close proximity to larger vehicles, which diminishes their visibility and increases their vulnerability to accidents. This heightened susceptibility is compounded by the fact that riders are in an exposed position, in stark contrast to the protection offered by enclosed vehicles. Moreover, the speed and agility of motorcycles and scooters, while essential for timely deliveries, can also result in more severe injuries in the event of a collision. Furthermore, a study from Australia indicated that engaging in delivery riding is an occupation fraught with notable road safety hazards, primarily driven by the pressures of the job (for example, time pressures), which often lead to the adoption of risky behaviors [37]. According to a University College London survey, 47% of delivery drivers reported driving above the speed limit, 30% reported driving through a red light being under time pressure, and 41% reported using a navigation app, which created a distraction [38].

In Israel, since 2016, it has been illegal for E-bikes/M-scooters to use sidewalks. These users should use specialized bike paths, but if such paths are unavailable, they should share the road with motorized vehicles [39]. Collisions between an e-tool and a motorized vehicle contributed to more severe injuries, higher rates of head trauma and severe head injuries, and poorer hospitalization outcomes [40,41,42,43]. Due to a quarter of injured children and adolescents working in delivery roles during the night, it becomes evident that breaching the law prohibiting nighttime employment for youth results in one of the most severe injury mechanisms—RTCs. Nighttime driving poses greater risks than daytime driving due to factors such as fatigue, decreased visibility, and changes in vision [44].

Helmet use must be made mandatory and enforced for these riders to reduce the risk of head injuries in the event of a collision [41,45]. Moreover, it is imperative for authorities to develop dedicated lanes for these travelers. In areas where such lanes are unavailable, it is crucial to enhance motor-vehicle drivers’ awareness of small electric devices on the road that may be challenging to detect visually or audibly [46]. In addition, tailored media campaigns should educate road users about the dangers of using headphones and mobile phones while riding [39].

In the food industry, where a significant number of children and teenagers are employed (the second largest employment industry of children), it is evident that the prevalence of severe injuries is comparatively lower, with most cases involving cuts. However, it is crucial to underscore the potentially life-altering nature of certain injuries, particularly burns in this industry, which have been identified in the literature as an “underappreciated public health hazard” [47]. Burns, though less frequent, can have profound and lasting consequences if not addressed promptly and effectively. It is worth highlighting that a significant presence in the group of those working at night, despite the prohibition on employing children and adolescents, was from the food industry. It is well-established that working in the evening and overnight hours exposes employees to an elevated risk of workplace accidents and subsequent injuries [48].

Assessing work environments is crucial to identifying potential hazards and preventing them, as each specific location within the food industry may have its unique risks. For instance, implementing simple and effective measures such as using cut-resistant mesh gloves and dedicated containers for broken glass can prevent cuts in the food industry. Providing non-slip, closed-toed shoes at no cost can reduce the risk of slips and falls. Additionally, using protective gloves when handling hot pots or working with hot, deep-frying oil, along with employing barriers, guards, or enclosures to prevent contact with hot surfaces, can help prevent burns in the food industry. Furthermore, here once more, the rigorous enforcement of child labor laws is imperative, particularly to ensure that children are not employed in nighttime work shifts. This demands vigilant monitoring and inspection by labor authorities to detect violations. Employers must be held accountable with swift and decisive penalties for non-compliance to deter the exploitation of child labor.

To cultivate a generation of safety-conscious workers, the groundwork must begin early by raising awareness among parents and communities. To ensure that young people understand potential workplace risks, education on workplace hazards, risks, and workers’ rights should be introduced in schools and continue through vocational training and apprenticeship programs. Employers, including formal and informal enterprises and family businesses, should receive guidance on the specific risk factors that young workers face, as well as insights into work tasks and conditions suitable for various age groups. Lastly, the support and representation provided by workers’ organizations become crucial as young individuals embark on their professional journeys, enabling them to assert their rights and voice their concerns.

## 5. Limitations

It is important to recognize the constraints of the present study. While the trauma registry encompasses all Level I trauma centers, it includes 14 out of 20 Level II trauma centers. These Level II trauma centers are not part of the registry and handle approximately 5% of severe trauma cases. Consequently, there is a potential for underestimating the true extent of trauma; however, this is considered acceptable since the majority of cases are accounted for.

The process of searching free-text responses to define work types proved to be less effective when applied to girls, primarily due to a significant proportion of work types that were challenging to categorize. Future research endeavors should prioritize the exploration of gender differences in defining work types to enhance the validity of findings for both sexes.

Additionally, it is important to note that the study lacked information regarding the specific rates of employment among Jewish and Arab children, rendering it impossible to calculate hospitalization risks based on the actual number of employed individuals. Consequently, our analysis relied on the total population of children and adolescents rather than the subset of those engaged in work activities, which may introduce a potential source of bias in the results.

## 6. Conclusions

In light of the findings presented in this study, several key recommendations emerge to address the critical issues surrounding youth employment safety. Creating secure employment opportunities for Arab young people and prohibiting the employment of children and adolescents in building and reconstruction within the Arab sector is of utmost importance. Secondly, for employers, it is vital to conduct stringent enforcement efforts, particularly at construction and reconstruction sites, to ensure strict compliance with regulations prohibiting the employment of children. Likewise, the enforcement against nighttime employment for children and adolescents should be pursued. Robust enforcement mechanisms will act as a deterrent and safeguard the well-being of young workers. Among policymakers, such as those within the Ministry of Labor and Welfare, there is a pressing need to develop and implement targeted initiatives in peripheral regions. These initiatives should aim to create safe and viable employment opportunities for children and youth, thus mitigating the risks associated with hazardous labor practices. Lastly, for the benefit of the children themselves, it is imperative to initiate educational programs within the school curriculum, beginning as early as high school, that encompass essential aspects of youth employment, including laws, safety regulations, rights, and basic first aid. This proactive approach will better prepare young individuals for future employment, equipping them with the knowledge and skills needed to navigate the workforce safely. By fostering a culture of safety, education, and compliance, we can collectively work towards a future where young workers are protected and empowered in their pursuit of meaningful employment.

## Figures and Tables

**Table 1:** Demographic, injury, and hospitalization characteristics of children and adolescents injured at work, by population group, 2015–2022.

Characteristics	Jews	Arabs	Total	
**n**	244	398	642	
Demographic characteristics				
Proportion among the study population	38%	62%	100%	
Age, median [IQR]	17.0 [16.0–17.0]	17.0 [16.0–17.0]	17.0 [16.0–17.0]	
Sex **, % of males	85.7	96.7	92.5	
Peripherality Cluster Percentile ** (%)				
Most peripheral (clusters 1–4)	23.1	58.3	44.4	
Peripheral (cluster 5)	9.1	21.1	16.4	
Central (clusters 6–7)	30.2	7.3	16.4	
Very central (clusters 8+)	37.6	13.3	22.9	
Accident characteristics				
Type of work ** (%)				
Food industry	24.2	10.1	15.4	
Building and reconstruction	11.9	40.2	29.4	
Delivery	29.9	1.3	12.1	
Agriculture	4.5	3.8	4.0	
Retail sector	4.5	4.5	4.5	
Garage	0	2.8	1.7	
Other and unknown	25.0	37.3	32.9	
Injury Mechanism * (%)				
Road Traffic Crashes (RTC)	37.7	12.6	22.1	
Cuts and lacerations	19.3	22.1	21.0	
Fall	11.5	24.6	19.6	
Strike by object	16.0	20.9	19.0	
Burn	8.2	4.8	6.1	
From machine	3.7	7.3	5.9	
Violence	2.0	4.5	3.6	
Unknown	1.6	3.3	2.6	
Work at night ** (%)				
At night (10 p.m.–6 a.m.)	27.9	12.8	18.5	
Other hours	72.1	87.2	81.5	
Injury Characteristics				
Injury Severity Score (ISS) (%)				
Mild (ISS 1–8)	82.0	73.3	76.6	
Moderate (ISS 9–14)	9.8	14.6	12.8	
Severe (ISS 16–24)	4.5	4.8	4.7	
Critical (25+)	3.7	7.3	5.9	
Hospitalization Characteristics				
Hospitalization in ICU (%)	6.1	10.1	8.6	
Length of Stay ***, median [IQR]	2.0 [1–5]	3.0 [1–5]	3.0 [1–5]	
Operation during the hospitalization (%)	40.6	40.5	40.5	

* *p* value of χ^2^ test < 0.05. ** *p* value of χ^2^ test < 0.001. *** *p* value of Mann–Whitney non-parametric test < 0.05.

**Table 2:** Odds for at least moderate injury, by demographic and injury characteristics.

Characteristics	n	ISS 9+ (n, %)	Model I *		Model II *	
	432	99 (22.9%)	Odds Ratio (OR)	95% CI	Odds Ratio (OR)	95% CI
Population Group						
Arabs	249	64 (25.7)	**2.9**	**1.4–6.3**	**2.8**	**1.2–6.4**
Jews	183	35 (19.1)	1	-	1	-
Peripherality Cluster Percentile						
Most peripheral (clusters 1–4)	164	37 (22.6)	0.8	0.4–1.6	1.0	0.5–2.0
Peripheral (cluster 5)	69	11 (15.9)	0.5	0.2–1.1	0.6	0.2–1.4
Central (clusters 6–7)	69	11 (15.9)	0.6	0.3–1.4	0.7	0.3–1.6
Very central (clusters 8+)	108	27 (25.0)	1	-	1	-
Type of work						
Building and reconstruction	189	42 (22.2)	1.8	0.8–4.3	1.1	0.4–3.1
Delivery	78	27 (34.8)	**8.8**	**3.3–22.9**	2.6	0.6–12.7
Agriculture	26	10 (38.5)	**4.8**	**1.5**–**15.3**	2.8	0.8–10.2
Retail sector	29	9 (31.0)	**4.1**	**1.3**–**12.6**	2.1	0.6–7.2
Garage	11	2 (18.2)	1.5	0.3–8.4	1.1	0.2–6.6
Food industry	99	9 (9.1)	1	-	1	-
Mechanism of Injury						
Road Traffic Crashes (RTC)	90	34 (37.8)	-	-	**14.4**	**2.5**–**81.6**
Fall	97	37 (38.1)	**-**	**-**	**14.8**	**4.1**–**53.0**
Burn	33	4 (12.1)	**-**	**--**	**5.0**	**1.1**–**24.5**
Strike by object	70	11 (15.7)	-	-	**4.1**	**1.1**–**16.8**
From machine	25	7 (28.0)	-	-	**8.5**	**1.8**–**39.8**
Other and unknown	16	3 (18.8)	-	-	4.5	0.6–31.7
Cuts and lacerations	101	3 (3.0)	-	-	1	-

* This analysis was conducted only among 432 cases with identifiable types of work. Bold values indicate statistical significance. Model I includes population group, peripherality cluster percentile, and type of work. Model II includes population group, peripherality cluster percentile, type of work, and mechanism of injury.

## Data Availability

All data supporting this study were obtained from the Israeli National Trauma Registry (INTR). Data cannot be shared following the restrictions defined by the Ethical Committee of the Sheba Medical Center.
